# Recurrent Erythema Nodosum Associated With Finger Millet–Blended Injera: A Case Report on a Potential Dietary Trigger

**DOI:** 10.1002/ccr3.71192

**Published:** 2025-10-06

**Authors:** Agete Tadewos Hirigo, Amanuel Anegagregn Bizuneh, Bedasa Addisu, Sisay Tesfaye

**Affiliations:** ^1^ School of Medical Laboratory Science, College of Medicine and Health Science Hawassa University Hawassa Ethiopia; ^2^ School of Medicine, College of Medicine and Health Science Hawassa University Hawassa Ethiopia; ^3^ Department of Medical Laboratory Science, College of Medicine and Health Sciences Debre Berhan University Debre Berhan Amhara Ethiopia

**Keywords:** allergy, erythema nodosum, fermentation, finger millet–blended injera, recurrence

## Abstract

This case report presents a recurrent episode of erythema nodosum (EN) potentially associated with the consumption of finger millet–blended injera. The patient developed EN manifestations within 1 week of initiating intake of injera prepared from teff and finger millet, with symptoms persisting for 6 months. Following dietary elimination of finger millet, complete clinical resolution was observed, and no recurrence was documented during an 8‐month follow‐up. However, causality cannot be confirmed due to the lack of allergy testing, dietary rechallenge, and histopathological verification. This case highlights the value of detailed dietary assessment, including less commonly consumed cereals, in the diagnostic evaluation and management of recurrent or idiopathic EN.

**Summary**

- Finger millet may be an under‐recognized dietary trigger for recurrent erythema nodosum (EN).
- Clinicians should include dietary assessment in the evaluation of persistent or idiopathic cases.

## 1 Introduction

Erythema nodosum (EN) is the most common type of panniculitis, marked by painful and red nodules [1, 2]. It is often associated with various systemic conditions, such as infections, cancers, inflammatory bowel diseases, and pregnancy [2]. However, in more than half of cases, the cause of EN remains unknown [3]. EN typically appears symmetrically on the front of the lower legs but can also spread to the thighs, arms, and neck. These lesions rarely result in necrosis and typically heal on their own within 2–8 weeks without leaving scars. Systemic symptoms including fever, fatigue, and joint pain may also accompany EN [3, 4]. The exact pathogenesis of EN remains unclear; however, most evidence suggests the involvement of a type IV delayed hypersensitivity reaction to various antigens [5, 6, 7]. Regarding millet, one study reported that it can rarely induce severe anaphylactic reactions following ingestion. Among atopic bird keepers, 63% possessed millet‐specific immunoglobulin E (IgE), and all patients who experienced reactions after millet ingestion demonstrated millet‐specific IgE [7]. To date, no published reports have documented an association between EN and the consumption of fermented finger millet or millet‐blended foods as a potential trigger.

### 1.1 Case History and Examination

**FIGURE 1:**
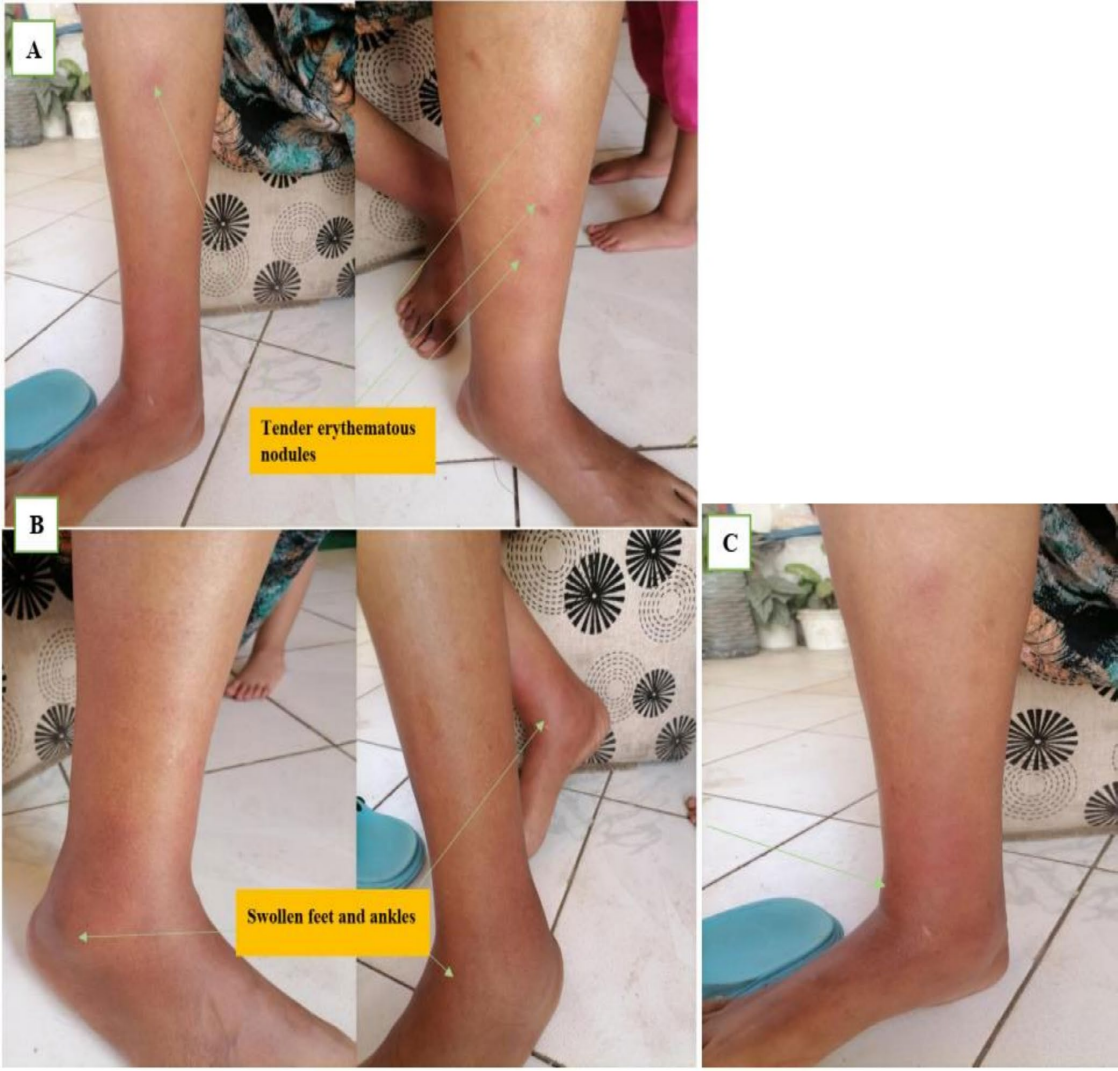
Tender erythematous nodules (A) and swollen feet and ankles (B and C) observed at the onset of Erythema nodosum and during the six‐month follow‐up period.

A 38‐year‐old woman with no underlying medical conditions presented with multiple tender, erythematous nodules on the bilateral lower extremities, specifically below the knees. The patient is a housewife, with no comorbidities, medication use, or documented allergies at the time of symptom onset. On December 22, 2023, the patient reported the onset of painful, tender nodules on both legs, accompanied by swelling of the ankles and feet, which significantly limited her ability to walk or stand for extended times (Figure 1).

### 1.2 Investigations, Differential Diagnoses and Treatment

Laboratory investigations were performed for the patient including complete blood count (CBC), prothrombin time (PT), activated partial thromboplastin time (aPTT), rheumatoid factor (RF), C‐reactive protein (CRP), liver function tests (LFT), erythrocyte sedimentation rate (ESR), and renal function tests (RFT). Qualitative tests for CRP and RF were non‐reactive. All other laboratory parameters were within normal limits, with the exception of a mildly elevated aPTT of 40 s (normal range: 22–38 s). The patient was initially misdiagnosed with cellulitis due to the presence of tender EN and was treated empirically with Cloxacillin 500 mg orally every 6 h for 7 days. She also had a mild cough, for which Azithromycin 500 mg once daily was prescribed for 3 days and the full course was completed. The clinical course and lack of response to antibiotics later supported a diagnosis of EN. The problem persisted, and 2 weeks later, the patient revisited the hospital. The internist prescribed a 3‐week tapering dose of Prednisolone along with Pantoprazole 40 mg daily to prevent gastric irritation. However, the patient developed significant epigastric discomfort and discontinued Prednisolone after 1 week. Despite this, her symptoms persisted and continued relapsing. She was subsequently treated empirically for intestinal helminthiasis with albendazole 400 mg once daily for 3 days. However, the painful red nodules on her extremities and the swelling in her ankles and feet persisted with recurrent episodes continuing until the end of June 2024.

## 2 Outcome and Follow Up

Subsequently, she consulted with a nutrition professional who inquired about any changes in her dietary habits, focusing on recent changes in food intake and the consistency of her nutritional habits. She reported that beginning approximately 1 week prior to the onset of EN, she had started consuming fermented flatbread (Injera) made from a blend of red Teff and finger millet flour twice a week. She maintained this dietary practice throughout the symptomatic period, which persisted for 6 months until the end of June 2024. The symptom of EN was resolved following consultation with a nutrition professional, who advised the exclusion of finger millet from her diet while continuing the consumption of injera made solely from teff or in combination with other grains. During the 8‐month follow‐up period from July 2024 to the time of this report, no recurrence of EN was observed, and she had no accidental re‐exposure to millet since the end of June 2024.

## 3 Discussion

Ethiopia is the second‐largest producer of finger millet in the world, following India, and is the leading producer in Africa [8]. In Ethiopia, finger millet is used to make flatbread (injera), malts for local alcoholic drinks, and non‐alcoholic drinks [9, 10]. Millet is an underutilized crop with significant health benefits, including low to medium glycemic index, high fiber content, polyunsaturated fatty acids (PUFA), and being gluten‐free [11]. it is rich in calcium, iron, and essential minerals like zinc, potassium, magnesium, and manganese, and also contains beneficial phenolic compounds and essential amino acids (e.g., cystine, methionine, and tryptophan) [12, 13]. Its consumption offers positive effects on cardiovascular health, obesity, and gastrointestinal function [14]. Additionally, due to its low to medium glycemic index, high fiber content, and gluten‐free nature, millet exerts antidiabetic effects and enhances insulin sensitivity [11, 15].

EN is a panniculitis characterized by red nodules, typically on the shins, with a multifactorial etiology that includes bacterial infections (such as tuberculosis and streptococcal), viral infections, systemic diseases (like sarcoidosis, Behçet's disease, and lupus), malignancies, medications, and vaccinations [16, 17]. The literature suggests that allergic sensitization to millet is primarily triggered by repeated inhalation of millet dust [6, 18]. Cross‐reactivity between millet and other grains, such as barley, oats, rice, and wheat, has been observed in certain individuals with millet allergy [6, 7]. All patients who experienced reactions after millet ingestion demonstrated millet‐specific IgE, and immunoblotting identified three major allergens in millet extract, indicating that millet is an important inhalant allergen in this population and that sensitization may subsequently trigger millet‐induced food allergy [7]. Otherwise, no cases were reported regarding EN initiated by finger millet.

Based on the clinical course, this case suggests that finger millet may act as a dietary trigger for EN in rare instances. Symptoms appeared approximately 1 week after the patient began consuming millet‐blended injera and persisted for 6 months, resolved completely after eliminating finger millet from the diet, with no recurrence during an 8‐month follow‐up. This pattern is consistent with a hypersensitivity reaction causing immune complex deposition in the venules of subcutaneous fat, triggering EN [19, 20]. As a limitation, causality is inferred rather than proven due to the absence of confirmatory allergy testing and histopathological analysis, as well as dietary rechallenge; nevertheless, finger millet may represent an under‐recognized trigger for recurrent EN. In addition, this report focused on a single patient, the absence of deliberate exposure to confirm EN, and limited generalizability to other populations. In conclusion, clinicians should consider dietary factors in persistent or idiopathic EN, particularly with rising millet consumption. Eliminating suspected allergens can resolve symptoms, highlighting the importance of dietary assessment in EN management.

## Author Contributions

**Agete Tadewos Hirigo:** conceptualization, data curation, investigation, methodology, resources, supervision, visualization, writing – original draft, writing – review and editing. **Amanuel Anegagregn Bizuneh:** data curation, formal analysis, investigation, writing – original draft, writing – review and editing. **Bedasa Addisu:** investigation, methodology, supervision, writing – original draft, writing – review and editing. **Sisay Tesfaye:** conceptualization, investigation, methodology, validation, writing – original draft, writing – review and editing.

## Consent

Written informed consent was obtained from the patient's parent(s) for the publication of anonymized information in this article.

## Conflicts of Interest

The authors declare no conflicts of interest.

## Data Availability

The authors confirm that the data supporting the findings of this study are available within the article.
